# Racial differences in breast cancer-specific mortality and CVD-specific mortality after breast cancer in post-menopausal women

**DOI:** 10.1186/s40959-025-00403-9

**Published:** 2025-12-17

**Authors:** Kerryn W. Reding, Alexi L. Vasbinder, Richard K. Cheng, Ana Barac, Yongzhe Wang, Warren J. Szewczyk, Reina Haque, Tarah J. Ballinger, Khadijah Breathett, Aladdin H. Shadyab, Regina Shih, Tomas Nuno, Robert A Wild, Xiaochen Zhang, Rami Nassir, Charles Mouton, Dorothy S. Lane, Lisa Warsinger Martin, JoAnn E. Manson, Marcia L. Stefanick, Michael S Simon, Veronica Jones

**Affiliations:** 1 School of Nursing, University of Washington, Box 357266, 1959 NE Pacific Street, WA 98195 Seattle, USA; 2 Public Health Sciences Division, Fred Hutch Cancer Center, Seattle, WA USA; 3 Division of Cardiology, School of Medicine, University of Washington, Seattle, WA USA; 4 Inova Schar Cancer and Inova Schar Heart and Vascular Institute, Falls Church, VA USA; 5 Breast Oncology Division, City of Hope, Duarte, CA USA; 6 Department of Research & Evaluation, Kaiser Permanente, Pasadena, CA USA; 7 Department of Health Systems Science, Kaiser Permanente, Pasadena, CA USA; 8 School of Medicine, Indiana University, Bloomington, IN USA; 9 Division of Cardiovascular Medicine, Indiana University Indianapolis, Indianapolis, IN USA; 10 Herbert Wertheim School of Public Health and Human Longevity Science, Division of Geriatrics, Gerontology, and Palliative Care, Department of Medicine, University of California San Diego La Jolla, San Diego, CA USA; 11 Rollins School of Public Health, Emory University, Atlanta, GA USA; 12 Department of Epidemiology and Biostatistics, Mel and Enid Zuckerman College of Public Health, University of Arizona, Tucson, AZ USA; 13 School of Medicine, University of Oklahoma, Norman, OK USA; 14 College of Medicine, The Ohio State University, Columbus, OH USA; 15 School of Medicine, Umm Al-Qura University, Mecca, Saudi Arabia; 16 John Sealy School of Medicine, University of Texas Medical Branch, Galveston, TX USA; 17 Renaissance School of Medicine, Stony Brook University, Stony Brook, NY USA; 18 George Washington University School of Medicine and Health Sciences, Washington, D.C., USA; 19 Division of Preventive Medicine, Brigham and Women’s Hospital, Harvard Medical School, Boston, MA USA; 20 School of Medicine, Stanford University, Stanford, CA USA; 21 Karmanos Cancer Institute, Detroit, MI USA

**Keywords:** Neoplasm - breast, Cardiovascular disease, Race, Mortality, Post-menopausal

## Abstract

**Background:**

Racial disparities in all-cause mortality after breast cancer (BC) have been documented. While elevated risk of BC mortality experienced by Black women is clear, it is unclear the relative contribution of cardiovascular disease (CVD) mortality to the survival disparity in Black women.

**Methods:**

This analysis from the Women’s Health Initiative (WHI) included 8,410 women diagnosed with invasive BC during follow-up. Cardiovascular (CV) events were defined as adjudicated myocardial infarction, heart failure, or stroke. Cause of death was determined through adjudication by medical chart review, ICD codes, death certificate, and/or autopsy report. 10-year cumulative incidence rates were calculated for CV events, CVD mortality, and BC mortality, stratified by race. Sub-distribution hazards ratios (sHR) were calculated using Fine and Gray models to account for competing risks.

**Results:**

In BC survivors (mean age = 70.9 years, median follow-up = 15.1 years), 8.5% self-reported as Black. Compared to White women, Black women had higher 10-year cumulative incidence of non-fatal CV events (10.9% vs. 8.2%, *P* = 0.001) and BC mortality (15.3% vs. 11.5%, *P* = 0.039). In contrast, White women had higher 10-year incidence of CVD mortality (7.2% vs. 10.1%, *P* = 0.001). BC mortality in Black women represented a higher proportion of death (35% vs. 20%), which was not true for White women.

**Conclusion:**

Our study reinforces prior findings that racial disparities are experienced by Black women with BC. This may be in large part driven by BC mortality. However, if improvements in BC mortality are made to reduce this gap, disparities in CVD mortality may become more prominent due to racial disparities in CV events.

## Background

Racial disparities exist in mortality after breast cancer (BC), with Black women experiencing higher BC mortality compared to White women [1]. More recently, research has shown elevated rates of cardiotoxicity and cardiovascular disease (CVD) mortality in Black women with BC [2, 3]. However, few studies have evaluated BC and CVD mortality simultaneously to understand which disease process is more responsible for the elevated overall mortality risk experienced by Black women with BC [2]. Elucidating racial differences in disease-specific mortality is vital to addressing racial disparities, particularly as risk factors may be shared as well as distinct across these mortality outcomes and may include social determinants of health (SDOH). The purpose of this analysis was to examine the racial differences in the 10-year cumulative incidence of CVD mortality and BC mortality among BC survivors from the Women’s Health Initiative (WHI).

## Methods

This cohort was comprised of women diagnosed with incident BC during their participation in WHI, a prospective study of post-menopausal women from 40 U.S. sites. All incident BC and incident non-fatal cardiovascular (CV) events (defined as a composite of incident myocardial infarction, heart failure, or stroke not resulting in death) were adjudicated through physician review of medical records; death from all causes were adjudicated through physician review of hospital records, autopsy report, and death certificate [4]. Detailed information on the definition of incident cancer has been published by Paskett et al., and on the definition of CV events in the WHI has been published by Curb, et al. [4, 5] For example, classification for MI involved review of medical history, electrocardiogram (ECG) readings, and results of cardiac enzyme/troponin determinations from hospitalization records using standardized criteria, which determined whether an MI was definite or probable [4, 6]. This analytic cohort included 8,410 BC survivors (stage I-IV) self-identifying as Black (*n* = 719) or White (*n* = 7,691) [7].

Cumulative incidence function plots estimated the 10-year cumulative incidence of each outcome (non-fatal CV event, CVD mortality, and BC mortality) stratified by race. Breast cancer diagnosis date was defined as time zero. Censoring occurred at the time of last documented contact date for those lost to follow-up, or the end of the observation period (February 19, 2022), whichever occurred first; death due to other causes was treated as a competing risk in each model. Differences in cumulative incidence were determined using Gray’s test for analysis accounting for competing events, and log-rank for analysis that did not account for competing risks.

**Fig. 1:**
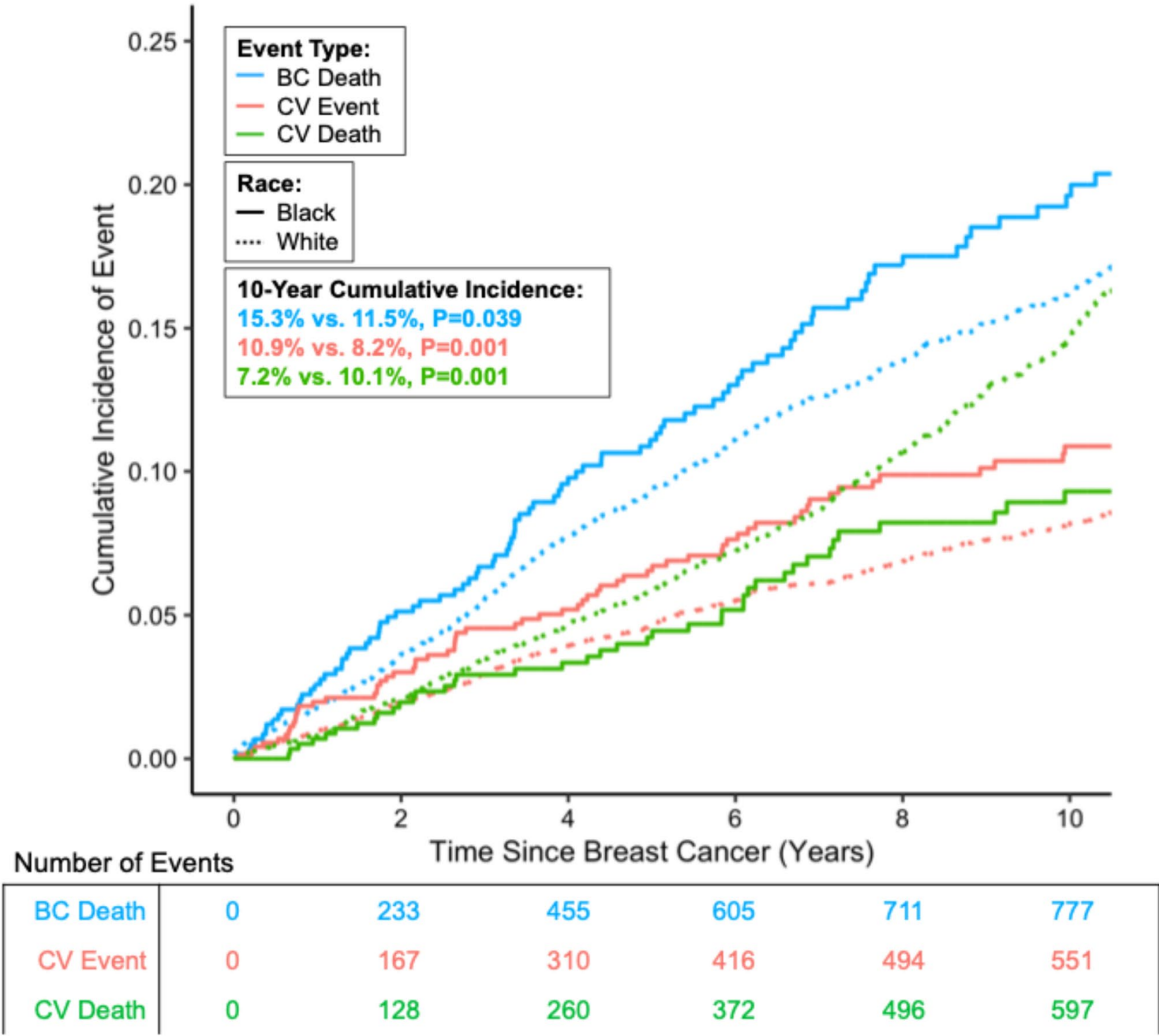
Cumulative incidence of CV events, CV death, and BC death stratified by race among post-menopausal breast cancer survivors. Cumulative incidences accounting for competing risks are shown in blue for breast cancer deaths, red for non-fatal CV events, and green for CV deaths in 8,410 women enrolled in the Women’s Health Initiative between 1993-1998 with a median follow-up of 15.1 years. Non-fatal CV events are defined as a composite of incident myocardial infarction, heart failure, or stroke not resulting in death. Abbreviations: BC, breast cancer; CV, cardiovascular

Sub-distribution hazards ratios (sHR) were calculated using Fine and Gray models to account for competing risk. The models were adjusted for factors selected a priori for their clinical importance, and their associations between race and each outcome. The factors considered for inclusion were age at diagnosis, self-reported income, BC stage, triple negative status, body mass index (BMI), alcohol consumption, physical activity, waist circumference, physical function, cigarette smoking, diabetes history, hypertension history, CVD history, age at menarche, parity, and age at menopause. Figure 1 details the confounders in each model. Secondary analyses explored confounding effects of BC treatment (e.g., radiation, chemotherapy) in a subset of participants for whom treatment data were available (60% of participants) due to their enrollment in the Life and Longevity After Cancer (LILAC) cohort [5]. Model assumptions were evaluated by Schoenfeld residuals tests; no violations were found. All analyses were performed in R 4.2.1. Statistical significance was determined using a threshold two-sided P-value < 0.05.

## Results

Participants had a mean age at BC diagnosis of 70.9 years (standard deviation [SD] = 7.8) (Table 1). Median follow-up was 15.1 years (Interquartile Range [IQR]: 8.4–24.3). 8.5% of women self-reported as Black or African American; 91.5% self-reported as White or Caucasian. Using SEER staging, the majority (75.8%) had localized stage, 22.7% had regional, and 1.5% had distant stage at diagnosis. At baseline, Black women were more likely to be diagnosed with distant stage (2.4% vs. 1.4%) and triple negative BC (13.2% vs. 6.6%), have higher BMI (32 vs. 28 kg/m^2^), history of diabetes (26.1% vs. 8.5%) and hypertension (74.7% vs. 48.0%), versus White women. No difference in age at BC diagnosis was detected. Black women experienced a 10.9% 10-year cumulative incidence of non-fatal CV events versus 8.2% in White women with BC (*P* = 0.001) (Fig. 1). Black women had a shorter median time to death for BC at 4.1 vs. 5.1 years observed in White women (*P* = 0.027), and a shorter time for CVD mortality of 7.4 vs. 11.1 years, respectively (*P* = 0.007). The leading causes of death during all years of follow-up were BC (*n* = 886), CVD (*n* = 822), other cancers (*n* = 633), and Alzheimer’s/Dementia (*n* = 299).

**Table 1:** Characteristics of the study sample

	Overall cohort (*n* = 8,410)	Died of CVD (*n* = 822)	Died of BC (*n* = 886)
Age at breast cancer, mean (SD)^a^	70.9 (7.8)	75.1 (6.8)	70.8 (8.2)
Race			
White	7691 (91.5)	764 (92.9)	786 (88.7)
Black/African American	719 (8.5)	58 (7.1)	99 (11.2)
Ethnicity			
Hispanic/Latina	197 (2.3)	11 (1.3)	16 (1.8)
Non-Hispanic/Latina	8210 (97.7)	811 (98.7)	869 (98.1)
Education			
High school diploma/GED or less	1546 (18.4)	151 (18.4)	163 (18.4)
School after high school	3126 (37.2)	334 (40.6)	346 (39.1)
College degree or higher	3680 (43.8)	331 (40.3)	370 (41.8)
Unknown	58 (0.7)	6 (0.1)	7 (0.8)
Family income			
<$20,000	1028 (12.2)	129 (15.7)	132 (14.9)
$20,000-$49,999	3619 (43.0)	416 (50.6)	390 (44.0)
$50,000-$74,999	1678 (20.0)	132 (16.1)	160 (18.1)
>$75,000	1763 (21.0)	115 (14.0)	168 (19.0)
Unknown	322 (3.4)	30 (3.6)	36 (4.0)
History of MI			
Yes	262	56 (6.8)	33 (3.7)
History of diabetes			
Yes	838 (3.1)	112 (14.8)	88 (10.0)
BC stage			
Localized	6275 (74.6)	646 (78.6)	370 (41.8)
Regional/distant	2034 (24.2)	170 (20.7)	477 (53.8)
Unknown	101 (1.2)	6 (0.1)	39 (4.4)
Triple negative status			
Yes	599 (7.1)	52 (6.3)	122 (13.8)

^a^N (percent), unless otherwise specified

Regarding BC mortality, Black women experienced a higher 10-year cumulative incidence than White women (15.3% vs. 11.5%, *P* = 0.039) when accounting for competing risks (i.e., other causes of death, including CVD mortality) and when not accounting for competing risks (13.0% vs. 8.6%, *P* = 0.001). In multi-variable models, Black women experienced a 28% higher risk of BC mortality compared to White women (sHR: 1.28, 95% CI: 1.02–1.61, *P* = 0.037) (Table 2).

**Table 2:** Adjusted subdistribution hazard ratios for the association between Black race and outcomes in post-menopausal breast cancer survivors

	**Overall cohort**		**LILAC cohort**	
	**sHR**^**a**^ **(95% CI)**	***P*** **-value**	**sHR**^**e**^ **(95% CI)**	***P*** **-value**
BC Death^b^	1.28 (1.02, 1.61)	0.037	1.43 (0.87, 2.33)	0.16
CV Event^c^	1.20 (0.95, 1.52)	0.12	1.38 (0.93, 2.04)	0.11
CV Death^d^	0.60 (0.46, 0.79)	<0.001	0.46 (0.28, 0.74)	0.001

^a^Subdistribution hazard ratios are presented for multivariable models comparing Black vs. White participants adjusted for outcome-specific confounders

^b^Model adjusted for age at diagnosis, cancer stage, triple negative BC, physical function, smoking, waist circumference, history of CVD, and physical activity for modeling the risk of BC death

^c^Model adjusted for age at diagnosis, physical function, hypertension, smoking, diabetes, history of CVD, and income for modeling the risk of CV events

^d^Model adjusted for age at diagnosis, hypertension, physical function, smoking, diabetes, history of CVD, physical activity, and alcohol for modeling the risk of CV death

^e^Models are adjusted for outcome-specific confounders described above in addition to receipt of chemotherapy and radiation in subset of participants enrolled in LILAC

The pattern was reversed for CVD mortality, such that Black women had a lower 10-year cumulative incidence of CVD mortality compared with White women (7.2% vs. 10.1%, *P* = 0.001), when accounting for competing risks (i.e., other causes of mortality) though not when competing risks were not considered (6.0% in Black women vs. 4.8% in White women, *P* = 0.30). In multi-variable models, Black women experienced a 39% lower CVD mortality than White women (sHR: 0.60, 95% CI: 0.46–0.79, *P* < 0.001). Thus, with respect to CVD mortality, the competing mortality of BC influenced the associations observed between CVD mortality and race.

In a subset of participants in whom BC treatment data were available, including radiation and cardiotoxic chemotherapies, the associations between race and outcomes were somewhat stronger, though some did not remain statistically significant due to the reduced statistical power (Table 2).

## Discussion

Our study reinforces and is consistent with prior findings describing racial disparities in BC survival, with an approximately 30% elevated risk of BC mortality for Black women [1], and extends prior work by delineating the mortality outcomes contributing to the racial disparity among post-menopausal women. The elevated BC mortality observed in Black versus White women is of similar scale as shown by the American Cancer Society [1]. In the WHI’s nationwide cohort of post-menopausal BC survivors, BC mortality and CVD were the top two causes of death. CVD mortality represented 20% and 23% of all deaths in Black and White women, respectively, which was overshadowed by BC mortality in Black women (accounting for 35% of deaths), though not in White women (24% of deaths). The statistically higher risk of CVD death in White women is somewhat surprising given the higher risk for CV events in Black women seen in our study and in the literature [2]. That the risk of CVD death in White women was higher than in Black women despite the higher cumulative risk of CV events in Black women may be explained by the disproportionately higher BC mortality seen in Black women, underscoring that cancer outcomes are driving the survival disparity in this study population.

Interestingly, while the association between CVD mortality and race was influenced by the competing risk of BC mortality, the converse was not true. This is conceivably explained by the shorter time to BC mortality for Black women, and the lag in time between CV in Black women (red line in Fig. 1) and CVD mortality, during which time BC mortality emerged as a competing risk. Thus, our findings suggest that BC mortality may be attenuating the risk for CVD mortality in Black women. This is consistent with a report in the literature of BC survivors from the Maryland Cancer Registry showing that Black women with CVD had an elevated risk of BC mortality compared to White women but that Black women with CVD were not at a similarly elevated risk of CVD mortality [3]. The study based on the Maryland Cancer Registry which was comprised of pre- and post-menopausal women did, however, show an overall elevated risk of CVD mortality for Black women, finding that the risk was greatest for women aged 50–59 years [3]. 

Contributors to racial disparities in BC survivors are multi-faceted, likely representing SDOHs, including structural factors of racism. As one example, race-based corrections have been used in algorithms, such as BC risk assessment tools [8]. The use of race-based corrections results in lower estimations of BC risk in Black women, which can result in lower screening rates. This can subsequently lead to a greater proportion diagnosed at a later stage, which was seen in this study population, with an albeit small absolute difference but 71% higher proportion in Black (2.4%) vs. White women (1.4%). This then leads to higher BC mortality and the need for more aggressive treatment, which are associated with a higher risk of cardiotoxicity. In this study, while we detected late stage to be a factor in BC mortality, we did not detect stage to influence CVD mortality (data not shown).

At the same time, triple negative BC (TNBC) is more common in Black women, which has a higher risk of metastasis and recurrence than hormone-positive BC and can be more challenging to treat due to the lack of specific targeted therapies [1]. Thus, aggressive treatments, such as anthracycline chemotherapy, are more commonly required. While the reasons for the disproportionately high percentage of TNBC in Black women is unknown, potential explanations are again multi-faceted, encompassing such variables as genetic ancestry, reproductive factors and SDOH [9–12]. Complicating this further is the finding that Black women diagnosed with TNBC are less likely to receive surgery and chemotherapy than White women, which translated to a 28% increased risk of mortality due to BC [12]. With TNBC accounting for double the proportion of BC in Black vs. White women (13.2% vs. 6.6%) in this study, this could account for some of the elevated risk of BC mortality experienced by Black women; however, the elevated risk seen here persisted even after controlling for receptor status.

Cause of death can be challenging to discern. However, the WHI used physician-adjudication of hospital records, autopsy reports, and death certificates (in that order) to make a determination. This represents a gold standard, particularly when teasing apart BC mortality from CVD mortality in BC survivors [4, 13]. Study limitations included that we did not have data on cardiotoxicity (e.g., left ventricular ejection fraction [LVEF] decline) and had treatment data on only a subset of participants. In addition, this analysis was conducted in a cohort of post-menopausal women. Thus, findings may not be generalizable to younger women. However, given that our findings of a racial disparity in BC mortality are in broad agreement with that of published work [1], we are reassured by the representativeness of these findings.

In conclusion, our results showed that BC mortality is higher in Black women than White women, consistent with prior literature. Our results showing the difference according to whether BC mortality was vs. was not accounted for in the estimation of risk of CVD mortality indicates that BC mortality is a competing risk. Therefore, should the disparity in BC mortality be eliminated, it would be possible for racial disparities in CVD mortality to emerge because Black women would survive their BC to potentially experience greater rates of CVD mortality. This notion is supported by reports in the literature of elevated cardiotoxicity in Black women and a suggestion of a higher risk of a CV event after BC in Black women in this dataset. While some drivers of racial disparities are shared across outcomes (e.g., medical access to guideline-concordant care), some are distinct (e.g., CV medications) [2, 12]. Thus, there is a compelling need to comprehensively address the contributors to racial disparities for both CVD and cancer.

## Data Availability

Data are available in accordance with policies developed by the National Heart, Lung, and Blood Institute and Women’s Health Initiative (WHI) to protect sensitive participant information and approved by the Fred Hutchinson Cancer Research Center, which currently serves as the Institutional Review Board of record for the WHI. Data requests may be made by emailing [helpdesk@whi.org](mailto: helpdesk@whi.org).

## Abbreviations

BC: breast cancer
BMI: body mass index
CV: cardiovascular
CVD: cardiovascular disease
IQR: interquartile range
kg: kilograms
LVEF: left ventricular ejection fraction
m: meters
SD: standard deviation
SDOH: social determinants of health
SEER: Surveillance, Epidemiology, and End Results
sHR: sub- distribution hazards ratios
TNBC: triple negative breast cancer
WHI: Women’s Health Initiative
